# Antidepressant medications in dementia: evidence and potential mechanisms of treatment-resistance

**DOI:** 10.1017/S003329172200397X

**Published:** 2023-02

**Authors:** Harry Costello, Jonathan P. Roiser, Robert Howard

**Affiliations:** 1 Institute of Cognitive Neuroscience, University College London, London, UK; 2 Division of Psychiatry, University College London, London, UK

**Keywords:** depression, dementia, antidepressants, Alzheimer's

## Abstract

Depression in dementia is common, disabling and causes significant distress to patients and carers. Despite widespread use of antidepressants for depression in dementia, there is no evidence of therapeutic efficacy, and their use is potentially harmful in this patient group. Depression in dementia has poor outcomes and effective treatments are urgently needed. Understanding why antidepressants are ineffective in depression in dementia could provide insight into their mechanism of action and aid identification of new therapeutic targets. In this review we discuss why depression in dementia may be a distinct entity, current theories of how antidepressants work and how these mechanisms of action may be affected by disease processes in dementia. We also consider why clinicians continue to prescribe antidepressants in dementia, and novel approaches to understand and identify effective treatments for patients living with depression and dementia.

## Introduction

Depression in dementia is common. Individuals with dementia are twice as likely as age-matched controls to develop major depressive disorder and up to one-third of all-cause dementia patients develop depression (Asmer et al., 2018).

**Fig. 1.**
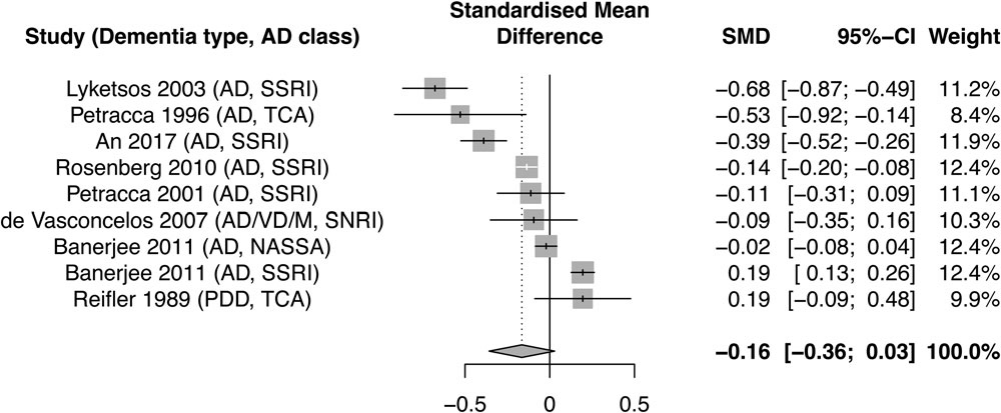
Reproduced forest plot of meta-analysis of included Cochrane review randomised controlled trials of antidepressants for depression in dementia. (Dudas et al. 2018) (AD - Alzheimer's dementia, VD - Vascular dementia, M - mixed dementia, PDD - Parkinson's disease dementia, SSRI - selective serotonin reuptake inhibitor, TCA - tricyclic antidepressant, SNRI - serotonin-noradrenaline reuptake inhibitor, NASSA - noradrenaline and specific serotonergic antidepressant).

Despite the widespread use of antidepressants in patients with dementia (Breining et al., 2017; Jester, Molinari, Zgibor, & Volicer, 2021) there is little evidence of therapeutic efficacy (see Fig. 1) (Dudas, Malouf, McCleery, & Dening, 2018). Though relatively few trials of antidepressants for depression in dementia have been conducted to date and most have studied depression in Alzheimer's dementia (AD), the largest and best quality studies have shown limited signal of any clinically meaningful difference (Banerjee et al., 2011). The lack of therapeutic effect cannot be explained by age effects as depressed older adults without dementia respond as well to antidepressants as other age groups (Gerson, Belin, Kaufman, Mintz, & Jarvik, 1999; Gildengers et al., 2002), though the effective dose range differs (Furukawa et al., 2019).

In fact, antidepressant use in dementia is not only ineffective but potentially harmful, as it is associated with increased risk of falls, hospitalisation and higher risk of mortality (Banerjee et al., 2021; Johnell et al., 2017).

Clearly further high-quality trials of antidepressants in dementia types other than AD are needed. To date only one randomised controlled trial (RCT) of a selective serotonin reuptake inhibitor (SSRI) in 14 patients has been conducted in Lewy body dementia (LBD) which did not show efficacy and found high overall burden of side-effects (Culo et al., 2010). Vascular dementia has similarly been neglected. Antidepressants are effective for post-stroke depression in patients without dementia (Li & Zhang, 2020). However, the overlap of vascular dementia with AD pathology (Attems & Jellinger, 2014) and evidence that higher vascular risk factor scores predict reduced response to antidepressants in late life depression (Sheline et al., 2010) does not suggest this patient group will buck the trend of a disappointing lack of efficacy of antidepressants in dementia. Even in frontotemporal dementia (FTD) where clinical use of SSRIs is widely advocated and seen as an effective therapy for depression and disinhibition in this patient group (Trieu et al., 2020), the evidence supporting its use is mixed (Huey, Putnam, & Grafman, 2006; Kaye, Petrovic-Poljak, Verhoeff, & Freedman, 2010). Most studies in FTD are open-label or case series designs, and the only RCTs of SSRIs (RCTs) found opposing results in terms of efficacy (Deakin, Rahman, Nestor, Hodges, & Sahakian, 2004; Moretti, Torre, Antonello, Cazzato, & Bava, 2003), though one trial of trazodone in 26 patients reported reduced depressive symptoms (Lebert, Stekke, Hasenbroekx, & Pasquier, 2004).

In the UK over a quarter of all people living with dementia take an antidepressant and the rates of prescription are increasing (Martinez, Jones, & Rietbrock, 2013). There is little evidence to support this rate of prescription and the lack of replication of the efficacy seen in depression without dementia could point to important mechanistic differences underlying depression in dementia.

Though there is evidence for the efficacy of some non-drug interventions in people with dementia (Orgeta, Qazi, Spector, & Orrell, 2015; Watt et al., 2021), therapeutic outcomes for patients with depression and dementia are poor (Fritze, Ehrt, Hortobagyi, Ballard, & Aarsland, 2011). Depression in dementia hastens cognitive decline, increases mortality, causes significant carer stress and accelerates admission to long-term care facilities (Asmer et al., 2018; Feng et al., 2010; Lavretsky et al., 2010; Rapp et al., 2011).

Substantial evidence supports the hypothesis that depression is an early prodromal symptom of dementia, commonly occurring up to 10 years prior to diagnosis, and a consequence of the distinct pathophysiology of individual dementia syndromes (Singh-Manoux et al., 2017). In cognitively normal older adults, biomarkers of Alzheimer's disease pathology are associated with subsequent depression and presence of depression accelerates cognitive decline (Babulal et al., 2016; Snowden et al., 2015). The underlying aetiology of depression as a prodrome of dementia is therefore likely to differ from depression in the general population.

Effective treatments for depression in dementia are urgently required. Understanding why standard antidepressants are unsuccessful in dementia has the potential to provide insight into both their mechanisms of action in typical depressed patients and the mechanisms driving depression in dementia, as well as aiding identification of new therapeutic targets.

In this review we summarise the reasons why depression in dementia may be a distinct entity, current understanding of how antidepressants work and how these mechanisms of action are affected by disease processes in dementia. We also discuss why antidepressants will continue to be used in dementia and the attendant clinical dilemmas faced by clinicians, patients and carers.

## A different syndrome of depression in dementia?

Depression is a heterogeneous and aetiologically complex syndrome. There are at least 256 possible unique symptom profiles that meet DSM-V criteria for a diagnosis of major depressive disorder (Buch & Liston, 2021). This degree of clinical heterogeneity has led to efforts to define subtypes based on symptom profiles (Buch & Liston, 2021). The reliability of different depression subtypes has not been established in the general population, largely due to transition between symptom profiles across and within depressive episodes (Lamers et al., 2012). However, this has led to studies highlighting how specific symptoms of depression respond differently to interventions and may predict overall response to treatment (Buch & Liston, 2021). For example, network analysis of individual depressive symptoms in antidepressant RCTs has identified improvement in low mood and insomnia as having greatest impact on subsequent remission and recovery (Komulainen et al., 2021).

Few studies have investigated how depressive symptoms differ across dementia types or in comparison with the non-dementia depression (Ehrt, Brønnick, Leentjens, Larsen, & Aarsland, 2006; Riedel, Heuser, Klotsche, Dodel, & Wittchen, 2010; Verdelho, Hénon, Lebert, Pasquier, & Leys, 2004). However, the clinical complexity of making a distinction between a diagnosis of depression from some of the other behavioural and psychological symptoms of dementia (BPSD) can be challenging as shown by the overlap between depression rating scales with other symptoms captured by BPSD measures (See Table 1). This raises the question of whether antidepressants are ineffective because they have been used to treat different syndromes.

**Table 1.** Depressive symptoms across seven depression rating scales that are also captured by measures of other BPSD symptoms (not depression items) on four BPSD measures

Depression symptoms across seven depression rating scales:	Co-occurrence of symptoms in four BPSD measures (not including depression items)			
	NPI	Behave-AD	CUSPAD	MOUSEPAD
Early insomnia	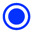	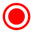	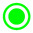	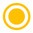
Middle insomnia	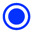	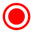	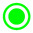	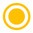
Late insomnia	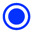	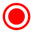	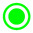	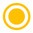
Hypersomnia	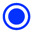	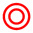	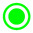	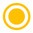
Sad mood				
Anxious	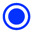	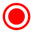	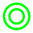	
Panic	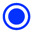	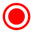	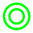	
Irritable	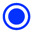	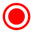	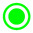	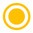
Mood reactivity	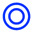			
Diurnal variation	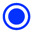	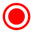	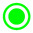	
Grief				
Appetite decrease	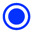		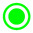	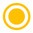
Appetite increase	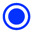		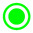	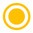
Weight decrease	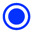			
Weight increase	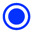			
Concentration	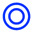	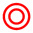		
Indecisiveness	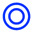	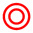		
Guilty	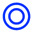		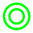	
Worthlessness		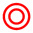	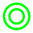	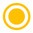
Pessimism				
Suicidal ideation				
Interest loss	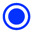			
Pleasure loss	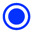			
Fatigue				
Energy loss				
Reduced libido				
Retardation	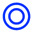			
Agitation	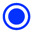	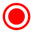	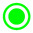	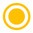
Somatic complaints	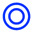	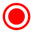		
Sympathetic arousal	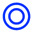	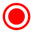	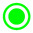	
Gastrointestinal	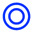	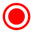		
Interpersonal sensitivity	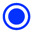	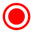	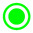	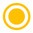
Leaden paralysis				
Past failure				
Punishment	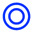	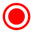	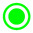	
Self-dislike				
Self-criticalness				
Crying	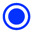	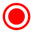		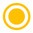
Lonely				
Effort	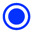			
Talked less	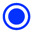			
People are unfriendly		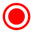	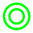	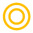
People dislike me	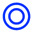	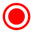	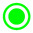	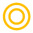
Feeling bothered	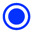	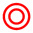	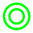	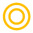
Feeling good				
Feeling happy				
Feeling needed				
Life is full				
Inner tension	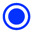	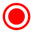	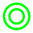	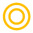
Inability to feel				
Hypochondriasis	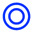	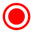	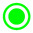	
Loss of insight	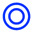	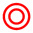	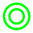	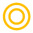

NPI, Neuropsychiatric Inventory (Cummings et al., 1994), Behave-AD, Behavioural Pathology in Alzheimer's Disease (Reisberg, Auer, & Monteiro, 1997), CUSPAD, Columbia University Scale for Psychopathology in Alzheimer's disease (Devanand et al., 1992), MOUSEPAD, Manchester & Oxford University Scale for the Psychopathological Assessment of Dementia (Allen, Gordon, Hope, & Burns, 1996).

Coloured circles for a symptom indicate that a scale directly assesses that symptom, whereas empty circles indicate that a scale indirectly measures a symptom. The seven depression rating scales are: Beck Depression Inventory (Beck, Steer, Ball, & Ranieri, 1996); Centre of Epidemiological Studies Depression Scale; Hamilton Rating Scale for Depression (Radloff, 1977); Inventory of Depressive Symptoms (Rush, Gullion, Basco, Jarrett, & Trivedi, 1996); Montgomery–Åsberg Depression Rating Scale (Montgomery & Åsberg, 1979); Quick Inventory of Depressive Symptoms (Rush et al., 2003); Zung Self-Rating Depression Scale (Zung, 1965). List of depressive symptoms adapted from ref – (Fried, Flake, & Robinaugh, 2022).

Studies of depressive symptom profile in dementia in long-term care facilities highlight how motivational symptoms such as apathy are common (Janzing, Hooijer, van ‘t Hof, & Zitman, 2002), and clinician-rated irritability is the most prevalent depressive symptom (Borza et al., 2015). However, both apathy and irritability are common in dementia in the absence of depression, and highly influenced by environmental factors (Jao, Liu, Williams, Chaudhury, & Parajuli, 2019).

Apathy affects 49% (Zhao et al., 2016) and 72% (Chow et al., 2009) of Alzheimer's disease (AD) and FTD patients respectively, markedly higher than that reported in older adults with depression without dementia (38%) (Yuen et al., 2015). Lesion studies have identified that the functional anatomy of apathy is associated with damage to fronto-striatal circuits (Levy & Dubois, 2006). Apathy also shares underlying neural and cognitive mechanisms with anhedonia, a core symptom of depression primarily characterised by a loss of interest in engaging in pleasurable activities. Motivational symptoms including apathy and anhedonia contribute to an ‘interest-activity’ cluster of depressive symptoms that predicts worse response to antidepressants in depression in the general population (Uher et al., 2012), but respond better than core mood symptoms to behavioural activation (Leontjevas et al., 2013). The presence of apathy in late-onset depression in mild cognitive impairment (MCI) also predicts subsequent development of AD (Ruthirakuhan, Herrmann, Vieira, Gallagher, & Lanctôt, 2019). This indicates that apathy is not only a common symptom in depression in dementia, but is central to the presentation of depression as a prodromal stage of dementia and poor response to antidepressant treatment.

Agitation is common in dementia affecting half of patients (Livingston et al., 2017), and is defined as inappropriate verbal, vocal, or motor activity that is not thought to be caused by an unmet need (Cummings et al., 2015). Irritability is a key component of agitation in dementia and strongly associated with depressive symptoms, both of which worsen with dementia progression (Volicer, Frijters, & Van der Steen, 2012). Though agitation is seen across psychiatric disorders, there is a clinical distinction between a patient with dementia and agitation consisting of wandering due to disorientation and a patient with agitated depression (Sampogna, Vecchio, Giallonardo, Luciano, & Fiorillo, 2020). It is likely that the prevalence of irritability as one of the most common symptoms in depression in dementia is due to this overlap, with agitation secondary to cognitive decline, and is a different entity to the irritability that characterises depression in the general population.

Few studies have assessed whether the phenomenology of depression in dementia is qualitatively different from depression in other populations (Ehrt et al., 2006; Riedel et al., 2010). However, apathy and irritability are examples of how key depressive symptoms in dementia, which are captured by most symptom scales, are also common in the context of BPSD without apparent depressed mood. This highlights just how challenging it is for clinicians to reliably diagnose depression in dementia and raises the question as to whether antidepressants are ineffective due to their being used to treat a different syndrome that isn't ‘depression’.

Due to the differing patterns of neurodegeneration across dementia subtypes it would be anticipated that the underlying mechanisms of depression, evolution of symptoms and response to treatment would be different in each disorder. However, few studies have investigated whether depressive symptom profiles or response to treatment differs across neurodegenerative disorders or stages of disease. Future research investigating the network dynamics of depressive symptoms over time could reveal how changes in specific symptoms are related, and which symptoms have the greatest impact on clinical improvement and treatment response.

Further trials of medications that treat depression as homogenous across dementia subtypes and stage of disease are likely to be ineffective. An alternative approach to understanding depression in dementia is to view it as a complex system of interacting depressive symptoms, which differ across dementia subtypes and stages of disease. Utilising this approach would promote tailored interventions for specific symptoms with greater importance at different stages of disease, development of depressive-symptom-network markers for each condition to guide interventions and diagnosis, and provide some mechanistic insights into the causes of depression.

## Neurodegeneration of monoamine pathways

The monoamine hypothesis of depression is over 50 years old (Coppen, 1967). It proposes that diminished activity in monoamine pathways (predominantly serotonin, noradrenaline and dopamine) plays a causal role in the development of depression. In the age of complex systems neurobiology and computational neuroscience, there is a consensus that such reductionist biochemical theories are over-simplifications (Cowen & Browning, 2015; Harmer, Duman, & Cowen, 2017). However, given that almost all effective antidepressants directly target monoamine system receptors or transporters, predominantly serotonin, monoamine function likely plays a central role in the therapeutic effect of antidepressants.

The monoamine systems project from small brainstem nuclei to widespread brain regions (Sasaki et al., 2008). Combined with an array of post-synaptic receptors, this anatomical distribution enables a wide variety of functions but is also susceptible to disruption from neurodegeneration (see Table 2). A potential reason for the lack of therapeutic effect of antidepressants in dementia would be disruption of the monoamine pathways needed to mediate their effect.

**Table 2.** Neurodegeneration of monoaminergic nuclei in dementia subtypes

Monoamine nuclei system	Alzheimer's dementia	Lewy body disease	Frontotemporal dementia
**Serotonin** (*Dorsal raphe nuclei* (*DRN*))	Phospho-tau neurofibrillary changes in the DRN occur early in AD (Braak stage I-II) and predate entorhinal pathology, one of the earliest brain regions affected by AD pathology (Grinberg et al., 2009)	In Parkinson's disease (PD) lewy bodies are found in the DRN and post-mortem studies have reported substantial loss of neurons in the DRN (Francis & Perry, 2007; Halliday et al., 1990).	A 40% reduction in DRN neurons is present at autopsy of patients with FTD, a similar level of degeneration to that seen in AD (Yang & Schmitt, 2001).
**Dopamine** (*Substantia nigra pars compacta* (*SN*) */ventral tegmental area* (*VTA*))	Neuronal loss in the SN and VTA in AD is highly variable. Over half of AD patients have neurofibrillary tangles in the SN, but <10% of patients have severe neuronal loss in this region (Gibb, Mountjoy, Mann, & Lees, 1989; Zarow, Lyness, Mortimer, & Chui, 2003).	Neuronal loss in the SN is a pathological hallmark of PD. Up to 80% of dopaminergic projections to the striatum from the SN are lost by the time of diagnosis (Cheng et al., 2010; Kish et al., 1988). Duration of illness correlates with loss of neurons in the SN (Cheng et al., 2010).	Though only mild atrophy has been reported in the SN and VTA in FTD, parkinsonism occurs in 30% of FTD patients (Mann, South, Snowden, & Neary, 1993; Park & Chung, 2013). One study found decreased striatal dopaminergic projections, and there are case reports of reduced pre-synaptic dopamine transporter binding in patients (Huey et al., 2006; Rinne et al., 2002).
**Noradrenaline** (*Locus coeruleus* (*LC*))	AD pathology occurs early in the LC, leading to severe loss of LC neurons (over 60%) (Grudzien et al., 2007; Zarow et al., 2003). Degeneration in the LC correlates with cognitive decline and has been associated with developing depression in AD (Grudzien et al., 2007; Zubenko, Moossy, & Kopp, 1990).	Neuronal loss in the LC is greater than the SN at post-mortem in PD patients. Over 80% of LC neurons are lost compared to controls (Zarow et al., 2003).	There are mixed findings of LC degeneration in FTD. One study reported that the LC is relatively spared in FTD (Yang & Schmitt, 2001). However, a larger post-mortem study found marked tau burden and neurodegeneration in the LC, which was greater in FTD-tau than FTD-transactive response DNA binding protein (TDP) (Ohm et al., 2020).

Serotonin is the monoamine that has primarily been implicated in the treatment of depression and is a target for most antidepressants, in particular SSRIs, the most widely used class of medications. The most direct evidence for serotonin's role in the pathophysiology of depression is from tryptophan depletion studies, in which transient reduction in serotonin synthesis is achieved via dietary depletion of its precursor amino acid, tryptophan. In recovered patients who are taking antidepressant medication, tryptophan depletion elicits a rapid worsening of depressive symptoms. However, tryptophan depletion has relatively little effect in healthy controls, currently depressed patients or even recovered patients who are unmedicated (Ruhé, Mason, & Schene, 2007).

The dorsal raphe nucleus (DRN) is one of two brain stem nuclei which are the primary sites of serotonergic innervation in the brain. The human DRN consists of ~ 250 000 neurons, 70% of which contain serotonin (Steinbusch, Dolatkhah, & Hopkins, 2021). Projections from the DRN are topographically organised rostrocaudally and innervate regions involved in mood regulation, motivation and memory, including the striatum, entorhinal cortex, hippocampus, pre-frontal cortex and amygdala (Steinbusch et al., 2021).

During the early stages of AD pathology (Braak stage I-II), the DRN is heavily affected by tau tangles (Rüb et al., 2000) and exhibits marked neurodegeneration, with estimates of up to 77% neuronal cells lost depending on disease stage (Lyness, Zarow, & Chui, 2003). As a consequence of the degeneration in the DRN, lower cortical serotonin, serotonin metabolites (5-hydroxyindoleacetic acid) and reduced serotonin transporter levels (Smith et al., 2017) have been reported in AD and other dementia syndromes (van der Zande et al., 2019). Degeneration in serotonergic pathways has also been implicated in the development of BPSD, including depression (Lanctôt, Herrmann, & Mazzotta, 2001; Vermeiren et al., 2015).

The noradrenergic system is a further target of antidepressants, including the serotonin–noradrenaline reuptake inhibitors venlafaxine and duloxetine. The locus coeruleus (LC), a small, pigmented nucleus located in the pons is the primary site for the synthesis of noradrenaline in the brain (Holland, Robbins, & Rowe, 2021). The LC projects widely and is implicated in modulating attention, memory, cognitive flexibility and sleep (Holland et al., 2021; Swift et al., 2018). Degeneration of the LC occurs early in AD and Parkinson's disease (PD) (Betts et al., 2019). Neuronal death of up to 30% of LC neurons has been reported in MCI, increasing to 55% in diagnosed Alzheimer's dementia (Kelly et al., 2017). Depletion of LC neurons in AD is more closely correlated with disease severity and staging than cholinergic degeneration in the nucleus basalis of Meynert (Holland et al., 2021; Kelly et al., 2017). LC degeneration has been implicated in agitation and irritability in AD (Liu, Stringer, Reeves, & Howard, 2018) and depression in PD (Wang et al., 2018). However, no study to date has examined whether LC integrity predicts antidepressant response in depression in dementia.

Human and animal studies have shown that dopaminergic transmission is crucial in motivated behaviour and reward processing (Le Heron, Apps, & Husain, 2018). Dopaminergic neurons project from the ventral tegmental area (VTA) and substantia nigra (SN) to the striatum (Sasaki et al., 2008). Dopamine function is closely related to other neuromodulators such as serotonin which it is co-released within the striatum (Zhou et al., 2005). This interplay with other neurotransmitters is believed to have an important role in cognitive functions and decision-making processes (Wert-Carvajal, Reneaux, Tchumatchenko, & Clopath, 2022) implicated in depression (see Table 3). Though not first-line and less commonly used, dopaminergic agents such as buproprion (a noradrenaline-dopamine reuptake inhibitor), and pramipexole (a dopamine partial agonist) are effective treatments in the management of depression (Papakostas, 2006).

**Table 3.** Examples of neurocognitive affective bias reported in depression and dementia

Affective domain	Negative bias in Depression without dementia	Negative bias in Dementia
Reward processing	Dysfunctional reward-processing, particularly in tests of option valuation, reward bias, and reinforcement learning (Halahakoon et al., 2020).	PD: impaired reward processing in option valuation, reward response vigour, and reinforcement learning. Reward processing overall was worse off dopaminergic medication (Costello et al., 2021). AD: Only reinforcement learning in AD has been studied to date, with clear deficits (Delazer, Sinz, Zamarian, & Benke, 2007; Sinz, Zamarian, Benke, Wenning, & Delazer, 2008; Torralva, Dorrego, Sabe, Chemerinski, & Starkstein, 2000). FTD: Evidence of impaired reward bias and reinforcement learning in behavioural variant FTD (Strenziok et al., 2011; Wong et al., 2019).
Memory	Preferential recall of negative emotional information in emotional word-based memory encoding tasks (Bradley, Mogg, & Millar, 1996). Impairments in executive function and memory are present in depressed patients and those with remitted depression *v.* controls (Rock, Roiser, Riedel, & Blackwell, 2014).	Progressive memory impairment characterises all dementia syndromes. Profile of memory deficits differs in dementia subtypes and stage of disease: AD at its core is an amnestic syndrome with early impairment in episodic memory followed by deterioration in word finding, spatial cognition and executive function (Karantzoulis & Galvin, 2011). In contrast FTD patients exhibit prominent early executive deficits and characteristic behavioural changes. However, overlapping memory deficits with AD in other domains occur (Ramanan et al., 2017). Other distinct profiles of cognitive impairment include greater deficits in semantic memory in VD *v*. AD (Graham, Emery, & Hodges, 2004). Few studies have investigated affective memory bias in dementia and there is conflicting evidence on whether memory in dementia is sensitive to affective content (Fairfield, Colangelo, Mammarella, Di Domenico, & Cornoldi, 2017; Maria & Juan, 2017).
Perception/Social cognition	Several studies report depressed patients categorise ambiguous facial expressions as more negative in the facial emotions recognition test (FERT) and have reduced sensitivity to happy faces (Roiser et al., 2012).	AD patients exhibit marked impairment in emotional perception of faces. This predominantly affects negative affective facial expressions, with poor detection and discrimination of anger, sadness and fear, compared to happiness (Elferink, van Tilborg, & Kessels, 2015). Interestingly this finding suggests a positive bias in dementia when compared to depression without dementia. Similar deficits in emotional perception have been reported in Lewy body dementia (LBD), PD and FTD (Elamin, Pender, Hardiman, & Abrahams, 2012; Heitz et al., 2016).
Emotional attention	Depressed patients exhibit impairments in affective attention and attentional shifting (Roiser et al., 2012; Taylor Tavares et al., 2007): In emotional Stroop tasks patients exhibit delayed naming of the colour of negative words (Broomfield, Davies, MacMahon, Ali, & Cross, 2007; Roiser et al., 2012). In an affective go/no go task depressed patients respond slower to happy targets (Erickson et al., 2005). However, negative attentional biases are dependent on duration of stimuli (Mogg, Bradley, & Williams, 1995), indicating that any bias could be driven by difficulty in disengaging from negative stimuli rather than overall attention.	Attentional deficits are prominent across dementia subtypes (McGuinness, Barrett, Craig, Lawson, & Passmore, 2010). However, research into emotional attention bias in dementia is limited: Among patients with AD there is no difference between positive and negative words on the emotional Stroop task (Doninger & Bylsma, 2007). However, in moderate AD significantly more errors were observed for negative words *v.* controls (Doninger & Bylsma, 2007). Delayed reaction times on the emotional Stroop task for both positive and negative words have been found in AD patients with depression *v.* without (Wang & Jia, 2007).

Dopaminergic dysfunction is associated with motivational symptoms of depression including apathy and anhedonia which are poorly responsive to antidepressant treatment (Husain & Roiser, 2018; Le Heron et al., 2018). Greater atrophy and loss of pre-synaptic dopaminergic projections to the striatum is associated with increased apathy and anhedonia across neurodegenerative disorders (Drui et al., 2014; Le Heron et al., 2018). VTA and SN neurodegeneration is most prominent in PD where up to 80% of dopaminergic projections to the striatum are lost by the time of diagnosis (Cheng, Ulane, & Burke, 2010; Kish, Shannak, & Hornykiewicz, 1988). This striking loss of dopaminergic neurons may explain why apathy and anhedonia are particularly common in PD, estimated to affect 40% (den Brok et al., 2015) and 46% (Lemke, Brecht, Koester, Kraus, & Reichmann, 2005) of patients, respectively. It may also explain why depressive symptoms in PD are poorly responsive to serotonergic and noradrenergic antidepressants (Troeung, Egan, & Gasson, 2013) but can respond to dopaminergic agonists used to treat motor symptoms (van der Velden, Broen, Kuijf, & Leentjens, 2018).

Whether involving serotonin, noradrenaline or dopamine, antidepressant mechanisms of action involve increasing monoamine levels in the synapse by preventing either metabolism or reuptake into the pre-synaptic neuron. However, as described above, there is significant degeneration of monoamine neurons in early dementia, leading to a loss of pre-synaptic innervation (see Table 2). Following the death of these neurons, monoamine release is markedly reduced, resulting in a loss of function in these systems which presumably reduces any impact of antidepressants.

In addition to loss of innervation, degeneration of pre-synaptic monoamine nuclei would also lead to loss of feedback inhibition. For example, the serotonin-1A (5-HT_1A_) autoreceptor controls serotonergic tone through feedback inhibition and has been hypothesised to lead to a reduction in synaptic serotonin levels early on in SSRI treatment (Fritze, Spanagel, & Noori, 2017). The time taken for 5-HT_1A_ autoreceptors to desensitise has been theorised to be an explanation of the delayed clinical effects of antidepressant treatment, and differences in 5-HT_1A_ receptor levels or have been associated with depression, and the response to antidepressants (Richardson-Jones et al., 2010). Though it is widely accepted that 5-HT_1A_ receptors participate in autoinhibition, their action is far more complex and the function of feedback is regionally organised and dependent on behavioural states (Altieri, Garcia-Garcia, Leonardo, & Andrews, 2012). Changes in 5-HT_1A_ distribution and binding have been reported in dementia but vary depending on brain region and dementia subtype (Elliott et al., 2009; Lai et al., 2003). For example, increased 5-HT_1A_ binding in the temporal cortex has been reported in vascular dementia (Elliott et al., 2009) but reduced binding in the same region has been found in AD which also correlated with higher agitation levels (Lai et al., 2003).

Understanding the relative deficits in monoamine function across dementia subtypes and stages of disease could guide which class of antidepressant may be most effective. However, as described above the dynamics and function of monoamine pathways are diverse, complex, and interdependent. There is considerable synergy between monoamine systems, for example co-release of dopamine and serotonin in the striatum, and innervation of the cholinergic system by the LC (Kelly et al., 2017; Zhou et al., 2005). Further research into the relationship between these systems and association with depressive symptoms would be needed to guide best use of existing therapies or development of novel interventions.

## Changes in neurocognitive processing

The cognitive neuropsychological model of depression suggests a causal role for negative affective biases in the development and treatment of depression (Roiser, Elliott, & Sahakian, 2012). Depression is associated with several types of negative affective biases (see Table 3) including changes in reward processing, negative perception of social cues, greater attention to aversive information and increased recall of negative relative to positive information (Harmer et al., 2017).

There is substantial evidence that antidepressants alter emotion and reward processing (Harmer et al., 2017; Roiser et al., 2012), thereby normalising negative affective biases. The cognitive neuropsychological model proposes that this modulation of core psychological processes in depression drives antidepressant therapeutic effect, explains why antidepressants take 4–6 weeks to improve depressive symptoms, and why they are most effective in conjunction with psychological therapy (Harmer et al., 2017).

Dementia is characterised by progressive cognitive decline and it is probable that the cognitive adaptations induced by antidepressants to normalise negative biases are not sustained due to these cognitive deficits. Neurodegeneration of core neural circuits involved in affective biases (in addition to the monoamine systems innervating them) may lead to further loss of antidepressant effects (see Table 2). For example, the reward processing circuit includes the ventral striatum, anterior cingulate cortex, amygdala and prefrontal cortex (Perry & Kramer, 2015). Serotonin co-release with dopamine in the ventral striatum is believed to be crucial in mediating the temporal dynamics of dopamine mediated reward prediction errors which drive reinforcement learning (Zhou et al., 2005). Activation in the ventral striatum is lower in both apathy and anhedonia (Husain & Roiser, 2018), and is directly altered by both serotonergic and dopaminergic medications (Admon et al., 2017; Stoy et al., 2012).

Degeneration of dopaminergic projections to the striatum is a core pathological feature of PD and LBD, with up to 80% of dopaminergic projections to the dorsal striatum lost by the time of diagnosis in PD (see Table 2) (Cheng et al., 2010; Kish et al., 1988). SSRIs such as fluoxetine bind at therapeutic doses in the ventral striatum and modify emotional valence and reward processing via changes in tonic and phasic activities of striatal neurons (Pasquereau et al., 2021). Loss of these neurons is therefore likely to impact the pharmacodynamics and therapeutic effects of antidepressants.

Other regions such as the amygdala, involved in processing emotional aspects of information and generating emotional reactions, project to the hippocampus which participates in the creation and maintenance of emotional associations in memory (DeRubeis, Siegle, & Hollon, 2008). More negative recall of information is a core negative affective bias found in depression, and antidepressant therapy has been consistently shown to be associated with activation of the amygdala (Nord et al., 2021). Prominent hippocampal and amygdala atrophy is a signature pattern of AD and FTD respectively (Barnes et al., 2006) and is associated with loss of emotional and behavioural regulation (Rosen et al., 2002). Neurodegeneration of these regions and other parts of the neural circuitry mediating affective biases is a likely key mechanism driving loss of therapeutic efficacy of antidepressants in dementia.

An alternative neurocognitive explanation for loss of antidepressant effect is that negative affective biases in depression in dementia are driven by different cognitive processes (see Table 3). For example, reward-related decision-making changes in AD have been found to be mediated by impairment in memory, inhibitory control and set-shifting rather than reward processing deficits (Perry & Kramer, 2015). Executive dysfunction, rather than reward valuation, has similarly been found to drive reward-based decision making in FTD (Perry & Kramer, 2015). In PD, reward processing impairment is dependent on dopaminergic medication state and subcategory of reward processing (Costello et al., 2021). These examples highlight how negative affective biases driving depression may have variable mechanisms which would not be targeted by existing antidepressant therapies.

## Loss of neuroplasticity

Almost all antidepressants increase the expression of brain-derived neutrophic factor (BDNF) via neutrophic tyrosine kinase receptor 2 (TRKB) (Casarotto et al., 2021). BDNF promotes neuronal maturation, plasticity and synaptic transmission by increasing production of brain cholesterol which facilitates cell membrane phospholipid fluidity of lipid rafts where many antidepressants interact (Casarotto et al., 2021). Antidepressants, including SSRIs and even rapid-acting agents such as ketamine, bind to TRKB, facilitating synaptic localisation and activation by BDNF. This induces synaptic plasticity that is believed to be a key underlying mechanism of antidepressants effects on mood (Casarotto et al., 2021). Mutation of the TRKB antidepressant binding site leads to loss of cellular, behavioural and neuroplasticity induced changes by antidepressants *in vitro* and in animal models (Casarotto et al., 2021).

Across different dementia disorders, BDNF is markedly reduced and has been implicated as a key pathological mechanism of neurodegeneration (Ng, Ho, Tam, Kua, & Ho, 2019; Zuccato & Cattaneo, 2009). Lower BDNF compared to healthy controls is seen in PD and AD, and worsens with disease progression (Zuccato & Cattaneo, 2009). Although, it remains unclear whether BDNF changes in PD and AD are a cause or consequence of disease, the first phase-I clinical trial of BDNF gene therapy for cognitive decline in AD commenced last year (Tuszynski, 2021). Substantial evidence supports a causal role of BDNF dysfunction in the pathogenesis of Huntington's dementia as the huntingtin protein plays a crucial role in the expression and vesicular transport of BDNF (Zuccato & Cattaneo, 2009).

Post-mortem studies have not supported a similar BDNF reduction in late-life depression compared to dementia (Nunes et al., 2018), and no study has yet investigated whether basal BDNF level predicts response to antidepressants. Further investigation of the effects of BDNF modulation on antidepressant response and mood symptoms in dementia is needed. It is likely that any BDNF-mediated therapeutic effect of antidepressants would be reduced in dementia due to marked reduction in BDNF levels. However, it could also be argued that if antidepressant efficacy is partly mediated by augmenting BDNF then strategies to increase BDNF levels could be an effective approach in dementia where it is lower.

Evidence suggests that post-synaptic modulation of calcium signalling is crucial for functional plasticity and regulation of the strength of synaptic transmission in response to neuronal activity via long-term potentiation or depression. Animal studies have shown SSRIs to protect against stress-induced synaptic neuroplasticity via direct blockade of voltage-activated L-type Ca^2+^ channels (Normann et al., 2018). Epidemiological evidence has found an association between L-type calcium channel antagonist use (e.g. verapamil hydrochloride) and reduced rates of psychiatric hospitalisation and self-harm in bipolar disorder (Hayes et al., 2019). This highlights how antidepressant function could be independent of their monoaminergic effects. Further investigation of the role calcium channel signalling has in mediating antidepressant effects is needed. However, this mechanism is also likely to be impacted by dementia pathology. Marked dysregulation of calcium homoeostasis is believed to play an important role in the development of AD neuropathology and hyperphosphorylated tau has been shown to directly impair L-type calcium channels (Stan et al., 2022; Wang, Shi, & Wei, 2017).

## Why antidepressants are still used in dementia: The clinical dilemma

Given the different aetiology and potential risks of treatment, it may appear straightforward to conclude that antidepressants have no role in depression in dementia and should not be used; however, this neglects the dilemma that patients, carers and clinicians face.

First, although trials have shown no superiority of antidepressants compared to placebo, the choice to prescribe antidepressants in clinical practice is not between antidepressant and placebo, but antidepressant and nothing. Though non-pharmacological interventions are effective and should be considered first-line treatments, many patients do not respond or are unable to engage with these interventions, which often require a good level of cognitive function. Even if only acting as placebo, we know placebo is effective at improving symptoms in both depression and in dementia (Hyde, May, Xue, & Zhang, 2017). Though clearly the use of antidepressants purely for their placebo benefit is unwise given the potential risks of harm and side effects, undoubtedly, this consideration will contribute to clinical decision making and represents an important reason why prescription of antidepressants in depressed people with dementia is continued.

Second, the lifetime risk of depression is high and it is estimated that up to half the population will experience one or more episodes of depression during their lifetime (Andrews, Poulton, & Skoog, 2005). When treating older adults with depression and dementia who have previously had episodes of depression successfully treated by antidepressants prior to developing dementia, the clinical question of whether to re-commence an antidepressant is difficult. Is this depression in the context of dementia or a relapse of existing major depressive disorder? Do antidepressants lose their efficacy in long-term depression users who develop dementia? Discontinuation of antidepressants in older adults with dementia who have remained on treatment for an extended period is similarly challenging, with evidence from one RCT showing worsening in depressive symptoms following discontinuation (Bergh, Selbæk, & Engedal, 2012).

Third, accurate diagnosis of depression in the general population is reliant on recall of depressive symptoms over the previous two weeks and detailed description of subjective symptoms by patients. In patients with dementia who have marked cognitive impairment this is often not possible, and clinicians will be reliant on collateral information from relatives and carers. However, the accuracy of such information varies enormously and is dependent on the nature of the relationship; it can also place considerable demands on family members who may be experiencing significant carer stress and low mood themselves. High turnover of staff working shift patterns in long term-care facilities (Costello, Cooper, Marston, & Livingston, 2020) can also complicate the process of making an accurate diagnosis and subsequent monitoring of depressive symptoms.

Finally, while antidepressants may not be effective in depression in dementia they are widely used for alternative indications which may overlap with depression. FTD guidelines support SSRI use to treat behavioural disinhibition and agitation despite no RCT having been conducted to date (O'Brien et al., 2017). Antidepressants are also used for treatment of neuropathic pain, which is highly prevalent in depression and dementia (Achterberg, Lautenbacher, Husebo, Erdal, & Herr, 2019), and they are frequently (mis)used to treat agitation as a substitution strategy to avoid antipsychotic prescription (Maust, Kim, Chiang, & Kales, 2018).

The clinical complexity of diagnosis and management of depression in dementia, and use of antidepressants for other BPSD, clouds how clinicians decide whether to prescribe antidepressants. We must be honest with patients and carers about the lack of efficacy and potentially harmful consequences of antidepressant use in dementia, but also acknowledge how challenging clinical decision-making regarding prescription in this patient group can be.

## Discussion

In this article we argue that antidepressants do not work in depression in dementia as it is likely a distinct entity that emerges as a consequence of a diverse array of pathological mechanisms, specific to each dementia syndrome, and which negates the therapeutic mechanisms of antidepressants. Antidepressants in depression without dementia have clinically meaningful small to moderate effects on improving clinical symptoms and preventing relapse (Cipriani et al., 2018). However, expecting the same therapeutic effects on a heterogenous syndrome in a patient population with varying degrees of neurodegeneration, pathophysiology and cognitive function may simply be wishful thinking.

In recent years considerable progress has been made in our understanding of antidepressant mechanisms of action which has shed light on the reasons why they may be ineffective in dementia. Though currently no effective pharmacological interventions for depression in dementia exist, there is reason to be optimistic.

It is unlikely that a single well-specified neurobiological cause of depression in dementia will be discovered. However, if viewed as a core component of dementia pathology, it offers a potentially more homogenous entity to treat with a clearer understanding of the temporal onset and markers of disease than depression in the general population. For example, depression in PD, which often progresses to PD dementia, is characterised by early degeneration of dopamine neurons in the SN and loss of dopaminergic projections to the striatum. Apathy and anhedonia, both disorders of motivation which share cognitive mechanisms, are prominent in depression in PD, correlate with loss of dopaminergic projections to the striatum and are responsive to dopaminergic medication. Utilising this understanding of the progression of pathology, phenomenology, and cognitive deficits within each disease could open the door for novel interventions, such as dopaminergic agents for depression in PD or deep brain stimulation targeting the striatum. This clear involvement of a distinct pathological process with a consistent trajectory is not present in depression in patients without dementia and demonstrates how understanding depression in dementia can give mechanistic insights into depressive symptoms.

Effective development of treatments for depression in dementia will depend on refining phenotypes and integrating our understanding of the evolution of depression over time with the progression in dementia pathology. A personalised medicine approach using clinical and biological markers to identify different phenotypes and effective treatments is an important area of future research in this patient group. Recent work using existing large cohorts has identified different trajectories of depressive symptoms in older adults depending on AD biomarker status (Banning, Ramakers, Rosenberg, Lyketsos, & Leoutsakos, 2021).

The processes discussed above of why antidepressants don't work in dementia offer insights and perspective for the future development of treatments for patients living with depression and dementia. Methods such as theory-driven computational modelling and approaching depression in dementia as a complex system of interacting behavioural, cognitive and neurobiological parts will be needed. However, these tools now exist and if integrated into future trials and drug development in depression and dementia could offer hope for identifying effective antidepressants for this patient group. The alternative of using the same approaches but expecting a different answer is unlikely to lead to the desperately needed treatments patients living with depression and dementia deserve.
